# Co-infection with hepatitis C virus (HCV) in Estonian intravenous drug users HIV epidemic

**DOI:** 10.1186/1471-2334-14-S4-O18

**Published:** 2014-05-29

**Authors:** Kristi Huik, Heli Rajasaar, Radko Avi, Merit Pauskar, Valentina Ustina, Kersti Kink, Lilia Novikova, Matti Maimets, Kai Zilmer, Irja Lutsar

**Affiliations:** 1 Department of Microbiology, University of Tartu, Tartu, Estonia

## 

Estonian concentrated HIV epidemic started in August 2000 when rare HIV-1 CRF06_cpx was introduced into the population of intravenous drug users (IDUs). The majority of these HIV-positive subjects are likely co-infected with HCV but the prevalence of HCV and its genotypes during HIV concentrated epidemic in Estonia is largely unknown. The Estonian HIV database collects clinical and laboratory data (including HCV) of HIV-positive patients on medical care. Of 4500 patients on medical care 3500 are entered into Estonian HIV database (in total 8664 diagnosed).

Aim: to describe the prevalence of HCV infection and the distribution of the HCV genotypes among HIV-positive subjects infected during HIV concentrated epidemic in Estonia.

Data for present analyses was extracted from Estonian HIV database on 2^nd^ of January 2014 and it comprised subjects diagnosed HIV-positive from 2000.

In total 2,420 of 3,476 (70%) HIV-positive subjects were HCV antibody positive and 1,184 (64%) were HCV RNA positive. More than half of HCV-positives were men (66%) and the median age was 32 years (inter quartile range 30-36 y). The prevalence of HCV was higher in subjects verified to be HIV-positive between 2002 and 2010 as compared to between 2011 and 2013 (90% – 60% in 2002 – 2010 vs 50% – 45% in 2011 – 2013; p <0.05). The prevalence of HCV-positivity was equally high in subjects reporting the use of intravenous drugs (88%), the use of other narcotics but intravenous drugs (69%) and persons who have not reported the use of illegal drugs (71%). In total 640 subjects had HCV genotype data available. The dominating genotypes were Ib (53%) and IIIa (36%), however, in recent years the prevalence of genotype Ia is raising (from 2.6% in 2005 to 25% in 2013). The distribution of HCV genotypes between different IDUs and non-IDUs was similar. Altogether 5% of HCV RNA positive subjects (66% with genotype Ib or IIIa; 29% with mixed or unknown; 5% with Ia or II) received HCV treatment and all except one admitted the drug usage. Of 59 subjects 48 (81%) received both HCV and HIV therapy.

Decreased prevalence of HCV-positive subjects among HIV-positives may suggest a lowered HIV transmission through intravenous route during the last years. The high prevalence of HCV in persons who did not report the use of illegal drugs might indicate under-reported drug usage in this population.

